# Digital Ergonomics of NavegApp, a Novel Serious Game for Spatial Cognition Assessment: Content Validity and Usability Study

**DOI:** 10.2196/66167

**Published:** 2025-04-02

**Authors:** Juan Pablo Sanchez-Escudero, David Aguillon, Stella Valencia, Mauricio A Garcia-Barrera, Daniel Camilo Aguirre-Acevedo, Natalia Trujillo

**Affiliations:** 1 Group of Epidemiology Universidad de Antioquia Medellín Colombia; 2 Grupo de Neurociencias de Antioquia University of Antioquia Medellín Colombia; 3 Mental Health Research Group Universidad de Antioquia Medellín Colombia; 4 Department of Psychology & Institute on Aging and Lifelong Health University of Victoria Victoria, BC Canada; 5 School of medicine Universidad de Antioquia Medellín Colombia; 6 Stempel College of Public Health and Social Work Florida International University Miami, FL United States; 7 Global Brain Health Institute University of California San Francisco, CA United States

**Keywords:** serious games, spatial cognition, digital neuropsychology, Alzheimer disease, content validity, usability

## Abstract

**Background:**

Alzheimer disease (AD) is the leading cause of dementia worldwide. With aging populations and limited access to effective treatments, there is an urgent need for innovative markers to support timely preventive interventions. Emerging evidence highlights spatial cognition (SC) as a valuable source of cognitive markers for AD. This study presents NavegApp, a serious game (SG) designed to assess 3 key components of SC, which show potential as cognitive markers for the early detection of AD.

**Objective:**

This study aimed to determine the content validity and usability perception of NavegApp across multiple groups of interest.

**Methods:**

A multistep process integrating methodologies from software engineering, psychometrics, and health measurement was implemented to validate the software. Our approach was structured into 3 stages, guided by the software life cycle for health and the Consensus-Based Standards for the Selection of Health Status Measurement Instruments (COSMIN) recommendations for evaluating the psychometric quality of health instruments. To assess content validity, a panel of 8 experts evaluated the relevance and representativeness of tasks included in the app. In addition, 212 participants, categorized into 5 groups based on their clinical status and risk level for AD, were recruited to evaluate the app’s digital ergonomics and usability at various stages of development. Complementary analyses were performed to identify group differences and to explore the association between task difficulty and user agreeableness.

**Results:**

NavegApp was validated as a highly usable tool by both experts and users. The expert panel confirmed that the tasks included in the game were representative (Aiken V=0.96-1.00) and relevant (Aiken V=0.96-1.00) for measuring SC components. Both experts and nonexperts rated NavegApp’s digital ergonomics positively, with minimal differences between groups (*r*_rb_ 0.08-0.29). Differences in usability perceptions were observed among participants with sporadic mild cognitive impairment compared to cognitively healthy individuals (*r*_rb_ 0.26-0.29). A moderate association was also identified between task difficulty and user agreeableness (Cramér V=0.37, 95% CI 0.28-0.54).

**Conclusions:**

NavegApp is a valid and user-friendly SG designed for SC assessment, developed by integrating software engineering and psychometric evaluation methodologies. While the results are promising, further studies are warranted to evaluate its diagnostic accuracy and construct validity. This work outlines a comprehensive framework for SG development in cognitive assessment, emphasizing the importance of incorporating psychometric validity measures from the outset of the design process.

## Introduction

### Background

Alzheimer disease (AD) is the leading cause of dementia, accounting for 60% to 80% of cases globally [1]. The rising number of AD cases represents a growing economic and social burden, challenging global health care and social systems [1]. Due to the absence of effective pharmacological treatments, preventive strategies targeting the preclinical stages of the disease have become the forefront approach to delaying the onset of symptoms [2]. Currently, the detection of preclinical AD relies on biomarkers obtained through techniques such as lumbar puncture and positron emission tomography [3]. Despite its accuracy, these methods describe limited scalability and utility in longitudinal population studies due to the associated costs, invasiveness, and potential side effects [4,5]. This is particularly problematic in low- and middle-income countries, where access to expensive diagnostic technologies is limited. Consequently, there is an urgent need for innovative, scalable, and noninvasive markers capable of detecting early cognitive or behavioral changes indicative of AD neuropathology [5,6].

Over the last decade, advances in cognitive neuroscience and software development have led to the emergence of multiple digital tools focused on detecting the earliest cognitive signatures of AD [7]. In this regard, recent evidence indicates that alterations in spatial cognition (SC) processes are present since the preclinical stage of AD. These deficits serve as promising indicators of the prodromal stages of AD, distinguishing affected individuals from healthy controls and marking disease progression [7]. Particularly, tests of spatial navigation applied using immersive virtual reality (VR) interfaces have been demonstrated to be sensitive and specific to the prodromic stage of the disease [7,8].

Although VR-based tests hold major potential, several limitations hinder their widespread adoption in clinical and primary care settings. Key barriers include the high costs of VR devices, the need for highly trained personnel to configure and operate these systems, and logistical challenges such as the time and physical space required for evaluations. For instance, many VR-based tasks necessitate the examinee walking several meters, which can be impractical in clinical environments [7]. The complex interfaces and reliance on unfamiliar control peripherals also present accessibility issues, particularly for older adults who may lack previous experience with such technologies. Together, these factors limit the scalability and feasibility of VR-based assessments for widespread, population-level implementation [4].

In response to these limitations, we have developed NavegApp, a serious game (SG)–based platform encompassing gamified tasks for assessing SC components [9]. This platform was developed by integrating game design principles and measurement properties analyses into the software development life cycle (SDLC), aiming to simplify digital interfaces while preserving adequate psychometric performance for the neuropsychological assessment of individuals at risk of developing neurodegenerative conditions, such as AD. The versatility and compatibility of NavegApp with various devices make it suitable for integration into different settings beyond research, such as primary health care services, nursing homes, or remote assessments. Such flexibility is essential in low- and middle-income countries, where access to specialized cognitive assessment or follow-up services may be limited [10].

Despite the foreseeable advantages, the development of a valid and reliable digital tool that integrates cutting-edge cognitive neuroscience with gamified interfaces into highly usable and ergonomic apps tailored to low- and middle-income countries face several challenges [11]. First, SGs must be precisely tailored for cognitive assessment, requiring a robust theoretical foundation for designing or adapting tasks and interfaces. Second, the SDLC must incorporate psychometric analysis, necessitating a strategic integration of software engineering and health measurement validation best practices. Third, creating an SG for individuals with varying levels of functionality demands careful consideration of digital ergonomics and usability [11]. The following sections present the key considerations and insights we used to address these challenges and implement NavegApp.

### SC as a Source of Cognitive Markers for AD

SC involves a range of cognitive functions, including the perception, organization, and use of location and object–based information to understand and navigate through physical or mental spaces [12-14]. When integrated, these processes facilitate complex spatial behaviors, such as solving mazes or tracing routes from point A to point B using landmarks or self-positioning as a reference [13]. Due to the complexity and extent of the brain areas supporting SC, neurodegenerative diseases, such as AD, affect this cognitive process in distinct and characteristic ways [12].

Accrued evidence suggests that subtle cognitive and behavioral deficits in SC can be detected during the preclinical and prodromal stages of AD, stemming from the effects of AD pathology in specific brain regions, such as the medial temporal lobe and parietal cortex [2,8,15-17]. People living with AD exhibit spatial impairments that intensify as AD pathology progressively spreads [12,15]. In the preclinical stage of AD, metabolic alterations and diminished connectivity in the entorhinal cortex, retrosplenial cortex, and precuneus contribute to difficulties with allocentric spatial navigation [8,15,18,19]. During the prodromal phase, increased neuronal loss and shrinkage of the hippocampus and inferior parietal cortex lead to impairments in path integration, route learning, egocentric navigation, and mental rotation tasks [12,20]. Furthermore, disruptions in glucose metabolism and gray matter loss, resulting from amyloid accumulation in the parietal lobe and posterior parietal cortex, affect spatial working memory and the integration of visual information [8,15].

Mentioned impairments can be detected by using different neuropsychological tasks, as has been previously outlined in the literature [15,16,21-24]. A decreased performance in allocentric spatial navigation tasks, such as the hidden goal task, has been described as an early potential cognitive marker for AD [25-27]. Additional evidence also suggests deficits in motor representation since the prodromal stage of AD, which leads to impairments in mental rotation tasks when compared to healthy, age-matched individuals [28,29]. Complementarily, individuals with mild cognitive impairment (MCI) exhibit decreased performance on the Corsi Block-Tapping test when compared to healthy control individuals, with more deficits observed in those with amnestic MCI and early AD, which suggests a decreased short-term spatial working memory [16,22-24].

To develop a valid and reliable instrument for cognitive assessment, a robust theoretical framework is essential [30]. Such a framework guides the formulation of hypotheses, interpretation of results, and design of tasks during the development of the assessment tool [31]. The SC model, as outlined by Jacobs [13], aligns with previous evidence by emphasizing the role of 3 key processes consistent with empirical evidence of SC deficits: the orientation of individual objects, as seen in mental rotation tasks; spatial orientation for self-navigation within large-scale environments, as seen in allocentric and egocentric spatial navigation tasks; and spatial memory, involving recalling information about object locations and constructing spatial scenes [13].

**Figure 1:**
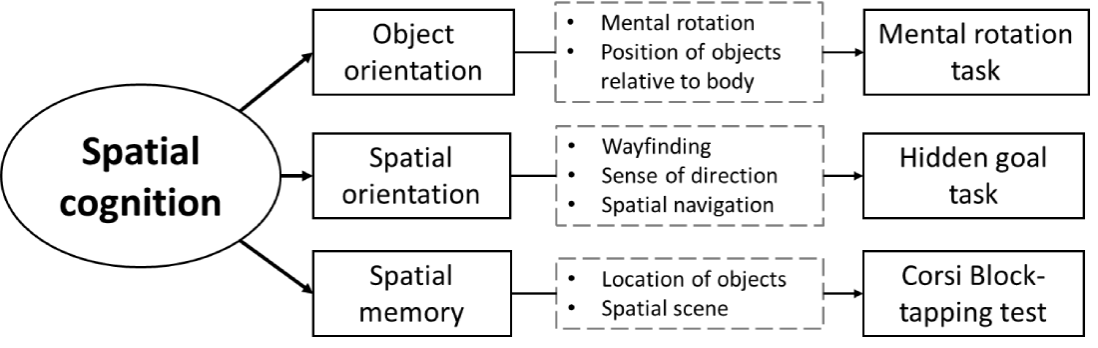
Spatial cognition framework and experimental tasks.

According to the model by Jacobs [13], these 3 components are interrelated, forming a dynamic network of cognitive abilities that can work synergistically to generate spatial representations or function independently to address specific tasks. This multifaceted nature of SC underscores that it is not a unitary construct and cannot be fully assessed through a single test [13]. Figure 1 illustrates the SC framework by Jacobs [13], and the corresponding neuropsychological tasks included in NavegApp.

Due to the alignment of the SC model by Jacobs [13] with the documented progression of SC deficits in AD, it provides a robust theoretical foundation for selecting and adapting tasks in NavegApp. By offering a comprehensive theoretical definition, the model enables the identification of key task components necessary for accurate construct measurement in psychometric terms. This approach ensures that core elements suitable for gamification are selected without compromising the measurement’s validity. Furthermore, while much research has focused on spatial navigation, the model by Jacobs [13] highlights the interconnected and multidimensional nature of SC components in forming spatial representations. This perspective underscores the importance of incorporating multiple metrics to ensure the instrument’s validity.

Although the use of SC measures in the detection and staging of AD holds promise, currently, there are no established protocols or tools for its application in clinical or research settings [12]. This gap presents an opportunity to develop a comprehensive evaluation system. However, creating such a tool requires carefully considering the adequacy of the SDLC to include relevant metrics for measurement purposes.

### Developing SGs for Cognitive Assessment

The SGs are games developed for purposes beyond entertainment [32]. These games integrate features of computerized tests and video games to create a relaxing and engaging experience for examinees while performing cognitive tasks designed to measure specific aspects of their cognition [33,34]. In recent years, SGs have proven valuable resources for cognitive and behavioral assessment in the context of neurodegenerative diseases [7,35]. Particularly, SGs enhance cognitive assessments by promoting optimal performance through increased engagement and motivation [35]. Furthermore, they enable precise and timely data collection and management, reducing the time required to derive insights from assessments and supporting the clinical decision-making process [33]. Consequently, specific recommendations for designing and adapting SGs for cognitive assessment tailored to individuals living with neurodegenerative diseases such as AD have been suggested [11]. These recommendations emphasize the importance of ergonomic criteria, usability, and measurement properties analysis during the SG development process [7,11,33].

Digital ergonomics involves design principles that create efficient, user-friendly interfaces [9,11]. Usability refers to the perceived ease and quality of use of a system, interface, or product and is a crucial aspect of the user experience [36]. Regarding the measurement properties, ensuring content validity—accurately representing the intended construct from the early development phase—is crucial for capturing essential and relevant information on cognitive performance [7,37].

Although the potential advantages of using video games for cognitive assessment at the earliest stages of AD are noteworthy, this approach remains underexplored and constitutes only a small proportion of the available tools [7]. One critical limitation is the lack of standardized frameworks incorporating evidence–based recommendations for SG development while integrating psychometric principles into the design process.

Most existing discussions and recommendations emphasize the benefits of designing immersive interfaces [38], improving user-interface interaction [39], and enhancing the overall user experience and engagement [40]. While these aspects are undoubtedly important, they do not guarantee the validity of the metrics produced by such tools [37]. Moreover, many instruments designed to measure core components of SC, such as spatial navigation and spatial memory, fail to provide detailed descriptions of their development processes, usability metrics, or feasibility analyses in at-risk populations or individuals in the earliest stages of AD [7]. This gap raises substantially concerns about potential spectrum bias, as the validity and generalizability of these tools may be compromised when applied to diverse populations [7].

The absence of a robust framework for developing SGs specifically tailored for cognitive assessment underscores the need for a comprehensive, transparent, and literature-supported approach. Such a framework should draw on principles from both software engineering and psychometrics to ensure the reliability, validity, and usability of these tools. Thus, developing an SG for cognitive assessment must adhere to software engineering best practices and design principles, particularly the SDLC [36]. This structured process outlines the step-by-step procedures for software development, covering design, development, testing, and maintenance stages, aiming to ensure efficient software development and deliver a high-quality product [36,41]. The process begins with design and development, enabling informed decision-making on system requirements and software architecture. The testing phase aims to ensure software functionality through iterative testing to address anomalies and incorporate user-desired features. This phase also allows collecting user experience data, including usability, digital ergonomics, and critical measurement properties. Once testing and verification are complete, the software is ready for deployment. In the context of cognitive assessment, software deployment typically marks the beginning of the clinical utility validation process [37].

In addition to considering the SDLC, incorporating gamification into SGs for cognitive assessment is equally important [40]. Gamification involves integrating game elements such as badges, counters, or leaderboards into tasks to enhance user engagement without compromising the validity of the assessment [32,37]. For example, graphic elements can be adapted in SC tasks to support a narrative and increase user engagement. However, the spatial relationships among these elements must be preserved to avoid altering task protocols or introducing performance biases.

Given the potential of SC measurements for early identification of individuals at risk for neurodegenerative diseases and the current lack of valid and user-friendly tools for scalable health care cognitive assessment services [9,11,38], this study introduces NavegApp, an SG-based platform designed to assess SC components. By leveraging the SC model by Jacobs [13] and incorporating software engineering best practices, this research seeks to produce a reliable and valid assessment tool that enhances early diagnosis and supports preventive strategies in diverse populations. For this, we focus on 3 critical metrics for SG implementation: content validity, digital ergonomics, and usability perception. Furthermore, this research addresses the gap in standardized frameworks for SG development by offering a detailed account of the development process, serving as a model for future work in the field. Using an iterative SDLC approach, we integrated evidence from cognitive neuroscience and psychometrics to create a fully functional, ready-to-use SG. Thus, the overall objective of this study was to evaluate the content validity, usability, and digital ergonomics of NavegApp as an innovative SG for SC assessment in the context of AD research. This contribution aims to advance tools for early AD detection and improve clinical outcomes through timely intervention.

## Methods

### Overview

We conducted a prospective observational study implementing a structured, multistep process integrating psychometric, usability, and digital ergonomics analysis into the SDLC [36,37]. This process consisted of 3 stages. The first stage, the prealpha phase, involved software requirement analysis, cognitive tasks gamification, software architecture design, documentation, and coding. The second stage, alpha testing, was focused on identifying and resolving general usability issues, ensuring data transfer integrity, collecting evidence on digital ergonomics and usability perception, and evaluating the app’s content validity through an expert panel [37]. The third stage, beta testing, aimed to assess the app’s usability by comparing the perceptions of different groups of interest, including caregivers (ie, health professionals and relatives of people living with dementia) and potential end users [42]. Integrating psychometric properties and usability considerations into the SDLC offered the flexibility to test various software versions within target populations. This approach allows for the evaluation of specific hypotheses relevant to software development and improvement.

### Participants

A total of 212 participants (n=143, 67.5% female participants) were recruited over 10 months using nonprobabilistic sampling methods. The sample included young, healthy adults; asymptomatic PSEN1-E280A mutation carriers; individuals with sporadic MCI or early dementia; and caregivers. Recruitment was conducted through various research projects involving individuals at risk of developing AD and their relatives. The cognitive status of participants suspected of having MCI or dementia due to AD was determined through comprehensive clinical examinations, while genetic status was confirmed via genotyping.

The clinical examination protocol encompassed neurological and neuropsychological assessments conducted by trained personnel with extensive experience evaluating this cohort. Examiners were blinded to participants’ genetic status during the evaluations to minimize bias. The classification of participants was based on the criteria established by the International Working Group on MCI. This classification incorporated the clinical dementia rating, the subjective cognitive complaints scale, and the Lawton-Brody Instrumental Activities of Daily Living scale. Cognitive impairments were evaluated through a neuropsychological examination that included the Consortium to Establish a Registry for Alzheimer’s Disease-Colombian version protocol, validated for use in the country [43,44], alongside assessments of memory, language, visuospatial skills, processing speed, and executive functions.

In addition, a panel of 8 experts with expertise in neurology, experimental psychology, and psychometrics was invited to conduct content validity analyses. The panel was selected based on their accomplishment of eligibility criteria (ie, experience in the field, graduate level, and disposition for participation). Young, healthy adults were enrolled from the university campus. Given the qualitative nature of the data required for testing feasibility and usability, nonprobabilistic sampling was deemed appropriate for developing NavegApp.

### Ethics Approval

This study was conducted in accordance with national ethical guidelines for research involving human participants, as outlined in resolution 008430 of 1993, which governs human research in Colombia. The study was classified as minimal risk, as it involved no invasive procedures or collecting sensitive information that could adversely affect participants. The protocol was reviewed and approved by the Ethics Committee of the School of Public Health (Act 21030002-00153-2022).

All participants provided informed consent before their involvement. The process included a comprehensive explanation of the study’s objectives and methods, presented in accessible language tailored to the participant’s understanding. For individuals with MCI or early dementia, relatives or caregivers were present during the consent process to ensure full comprehension. Their understanding and permission for the individual’s participation were confirmed. Participants’ privacy and confidentiality were protected by deidentifying all data before analysis. Data were stored in independent files on web servers equipped with security protocols to prevent unauthorized access. Participants received financial compensation equivalent to one day’s labor (approximately $15 USD) as recognition for their time and involvement. The study complied with the ethical principles of the Declaration of Helsinki and all applicable regulations.

### Procedure

In total, 5 distinct sample groups were enrolled during the validation of NavegApp. Initially, older adults with either MCI or early-stage dementia tested NavegApp as a proof of concept of the interfaces during the alpha-testing phase. The rationale for including such participants was to avoid a potential spectrum bias during the earliest step of the app development by ensuring that the instructions provided, and screen distribution were comprehensible to participants, even when they exhibited signs and symptoms of cognitive impairment [9]. Adjustments were made based on their qualitative feedback and performance.

Next, to analyze content validity, 8 experts were invited to assess the updated version of NavegApp. The information was collected using a 2-round iterative process, consistent with Delphi method recommendations [45,46]. In round 1, experts were asked to give their opinion on the relevance and representativeness of each task included in NavegApp, aligning with the selected SC framework [13]. In addition, their input on the software’s digital ergonomics was sought. Subsequently, experts were presented with an adjusted version of NavegApp and an anonymized summary of results from the first round [46]. Qualitative feedback was gathered to improve the software further. Experts were also allowed to evaluate the digital ergonomics of the adjusted version. Concurrently, a healthy sample of undergraduate students was recruited to gather their feedback on digital ergonomics. Furthermore, data on the overall difficulty and user-agreeableness perception of NavegApp were collected.

**Figure 2:**
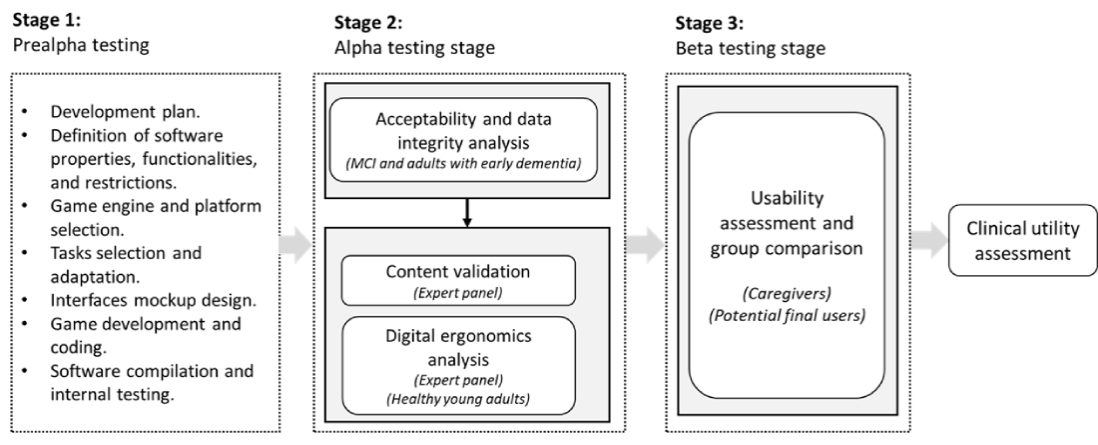
The workflow of NavegApp development and validation process. MCI: mild cognitive impairment.

In the subsequent beta testing stage, expert refinements and healthy young adults’ recommendations were incorporated into NavegApp. This phase involved enrolling 2 new groups of participants to evaluate the usability perception of the updated version. As advised by the expert panel, we recruited a separate sample of caregivers, including family members and health professionals affiliated with a Colombian dementia research institution, to test NavegApp and provide feedback on its usability. In addition, a sample of potential users, including individuals in the preclinical stage for familial AD (ie, PSEN1-E280A mutation carriers), individuals with sporadic MCI, and healthy adults, were invited to test NavegApp and provide feedback on its usability. Figure 2 provides a detailed description of NavegApp’s development workflow.

### Instruments

#### Computer System Usability Questionnaire for Videogame

The Computer System Usability Questionnaire for Videogame (VG-CSUQ) is an adapted version of the Spanish Computer System Usability Questionnaire (CSUQ) [42]. This version consists of 15 items rated on a 7-point Likert scale, ranging from “strongly disagree” to “strongly agree.” Factor analysis has shown support for a 3-factor structure consistent with previous literature, with factors including system usefulness, information quality, and interface quality [43,44,47-49]. The system usefulness factor assesses task learnability and completion ease, information quality gauges the clarity and user satisfaction with the game’s information, and interface quality pertains to the interface’s clarity and perceived quality [48]. In total, 4 scores were calculated following the prescribed scoring methodology, one for each usability factor and an overall usability score [43].

#### Digital Ergonomics Questionnaire

To evaluate NavegApp's digital ergonomics, an ad hoc questionnaire was tailored following the recommendations for SG design in the context of cognitive assessment for neurodegenerative diseases [11]. The questionnaire collects information on 8 ergonomic criteria for evaluating user interfaces. The full version of the questionnaire is present in Multimedia Appendix 1.

#### NavegApp

NavegApp is an SG designed as a narrative puzzle video game with 3 levels. Each level represents a specific component of the SC model by Jacobs [13], each tailored by gamifying tasks previously described as potentially sensitive to cognitive changes occurring since the preclinical stage of AD [17,22,28,50-55]. The tasks were adapted to a narrative video game format by applying gamification and game design principles aligning with the protocol description available for each one [39,44,48]. Thus, NavegApp includes a gamified version of a hidden goal task (gHGT) for testing allocentric spatial orientation [50], a gamified version of a mental rotation task used for testing the ability to manipulate objects in a 2D space [28], and a gamified version of the Corsi Block-Tapping test for the assessment of visuospatial short-term memory [54,55].

The gHGT comprised 8 trials, each requiring the participant to locate a hidden object (ie, hedgehog) that had been visible for 5 seconds. For orientation, participants could only use 2 distal landmarks. Within each trial, the starting position, spatial landmarks, and hidden goal occupied 1 of the 8 pseudorandom positions while maintaining the same mutual spatial relationships [50]. A tutorial was designed to ensure participants’ understanding of the task. An algorithm was implemented to detect unusual performance patterns, such as moving toward the landmark instead of the goal, excessively long paths, or extreme deviations from the goal, prompting a repeat of the tutorial if necessary.

The gamified mental rotation task consisted of 3 conditions (at 0, 90, and 180 degrees) with 16 trials each [28]. In each condition, participants were instructed to mentally rotate the object displayed at the center of the screen in a clockwise direction and subsequently select the correct stimulus from a set of 4 options. The stimuli were presented in the same order and positions as described in the original task, with visual and auditory feedback for correct and incorrect responses. To ensure comprehension of the instructions, a tutorial was implemented using a new stimulus, preserving the original task protocol as closely as possible.

Finally, the gamified Corsi block-tapping test asked the participants to repeat a predefined sequence of blocks in forward and backward sequence [54,55]. In the forward condition, participants were required to replicate the sequence in the same order as presented. In contrast, the sequence had to be reproduced in reverse order in the backward condition. For the gamified version, the participant must follow a mole through 9 holes located in the same position as the blocks from the standard in-person test [54]. The task’s stopping criteria were also preserved, ending after 2 consecutive failed attempts at sequences of the same length [54]. A counter displayed on the screen indicated the number of holes to explore in each trial, providing participants with a clear reference. In addition, visual and auditory feedback informed participants whether they successfully found the mole or failed the trial.

**Figure 3:**
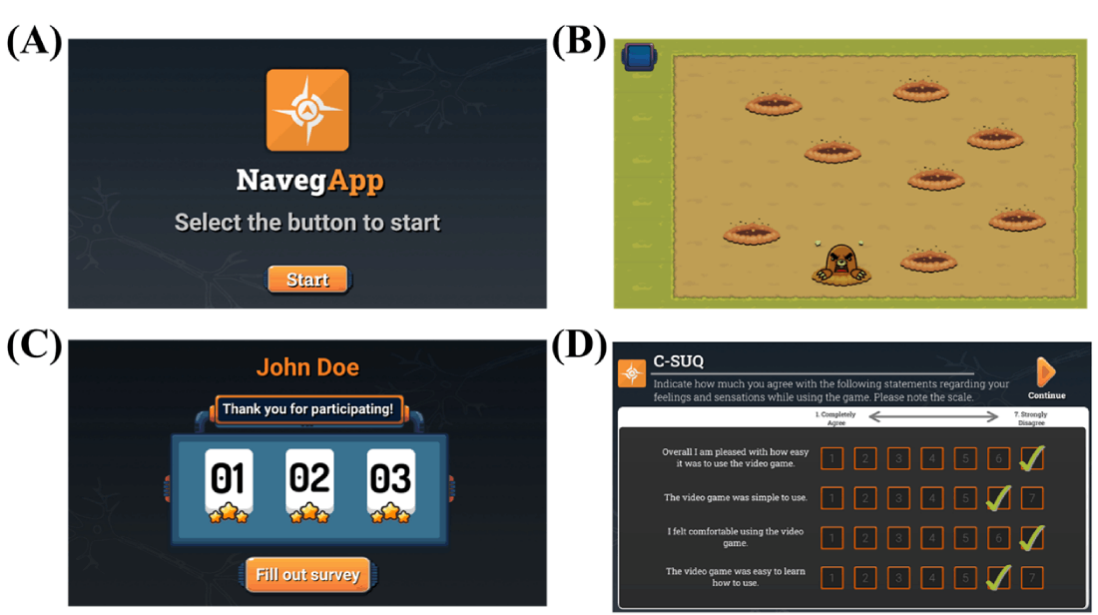
NavegApp’s interface screen captures.

Additional scenes were incorporated into the video game to capture essential data, including demographic information and the VG-CSUQ questionnaire. The Unity game engine (version 2022.1.20f1) [47] was used for developing NavegApp. Graphic material was obtained through memberships of 2D game assets in online stores. All the graphic material is protected by the Creative Commons (version 4.0) license. Figure 3 depicts example screenshots of NavegApp’s interfaces. The screenshots are presented in the English language for communication purposes. The original content was provided in the Spanish language, as it is the mother tongue of participants involved in the study.

### Statistical Analysis

Descriptive statistics were used to summarize the performance of formative and summative usability assessments in accordance with the recommendations for quantifying user experience [56]. Our approach adhered to established recommendations for usability and digital ergonomics testing for cognitive games [9,11,56,57], and content validation for measurement instruments in health settings [58-61]. Therefore, for content validity analysis, the experts’ opinion consistency was determined following the recommendations and procedures described by Yusoff [58] and Polit et al [59] during the alpha testing stage.

To account for the interrater chance of agreement, the modified κ statistic, based on the content validity index, was calculated [62]. This involved first computing the probability of chance agreement using the formula for a binomial random variable. The modified κ statistic was then derived using the proportion of agreements on relevance and the calculated probability of chance agreement [62]. Results were interpreted based on recommendations from the literature regarding the number of experts. Values >0.72 were classified as indicating good agreement, while values >0.88 were considered evidence of excellent agreement [62].

In addition, Aiken V coefficient [60] was calculated as a complementary measure of the experts’ degree of agreement regarding the relevance of the instrument elements using a Likert scale [60,63]. Unlike the content validity index, Aiken V accounts for the ordinal nature of ratings, making it particularly suitable for small samples of expert raters. This characteristic makes Aiken V an ideal measure for evaluating the content validity of instruments during early development stages [60,61].

The rank biserial correlation was used to estimate the magnitude of differences in digital ergonomics perception between experts and young, healthy adults testing the alpha version of NavegApp. In addition, the relationship between agreeableness and perceptions of difficulty was evaluated using the Cramér V coefficient.

The rank biserial correlation was calculated in the beta testing stage to evaluate differences in usability perceptions across participants. To evaluate differences in usability perception due to cognitive decline, the perception of sporadic MCI participants was compared against those of healthy adults in the remaining groups. A 95% CI was computed to estimate the precision of the effect size measures. All statistical analyses were performed using R (version 4.3.1; R Core Team) [62] and RStudio (version 2023.06.1; RStudio Inc) [64].

## Results

### Sociodemographics

The sociodemographic characteristics of the participants are presented in Table 1. All groups’ median years of education were more than 11 years, indicating that at least half of the participants had finished secondary school. According to the Colombian National Statistics Department, 87.2% (185/212) of the participants belonged to a low socioeconomic status, whereas 87.7% (186/212) were raised in urban areas [65].

**Table 1:** The sociodemographic characteristics of the participants.

		Alpha testing		Beta testing			
		MCI^a^ (n=16)	Healthy young adults (n=20)	Caregivers (n=22)	PSEN1-E280A mutation carriers (n=81)	Healthy controls (n=59)	MCI (n=14)
**Sex, n (%)**							
	Female	11 (69)	10 (50)	15 (68)	58 (72)	39 (66)	10 (71)
	Male	5 (31)	10 (50)	7 (32)	23 (28)	20 (34)	4 (29)
**Age (y)**							
	Mean (SD)	72.8 (6.16)	24.2 (6.31)	64.7 (12.7)	31.3 (8.36)	33.7 (10.5)	64.9 (7.59)
	Median (IQR)	75.5 (8.3)	22.0 (2.3)	71.5 (19.5)	31.0 (15.0)	34.0 (17.0)	65.0 (8.3)
**Years of education (y)**							
	Mean (SD)	11.0 (5.34)	12.5 (2.31)	12.2 (4.82)	11.6 (3.31)	11.5 (3.66)	12.9 (4.34)
	Median (IQR)	13.0 (8.0)	12.0 (2.3)	12.0 (5.0)	11.0 (2.0)	12.0 (2.0)	13.5 (4.8)
**Upbringing area, n (%)**							
	Rural	1 (6)	2 (10)	1 (4)	15 (18)	7 (12)	0 (0)
	Urban	15 (94)	18 (90)	21 (96)	66 (82)	52 (88)	14 (100)
**Socioeconomic status, n (%)**							
	High	0 (0)	0 (0)	8 (36)	2 (3)	1 (2)	4 (29)
	Low	16 (100)	20 (100)	10 (46)	78 (96)	55 (93)	6 (43)
	Medium	0 (0)	0 (0)	4 (18)	1 (1)	3 (5)	4 (29)

^a^MCI: mild cognitive impairment.

### Alpha Testing Stage: Data Integrity and General Acceptability

During the alpha testing stage, several issues were identified concerning the integrity of the data uploaded to the server. In total, 3% (1/36) records of task performance was not uploaded due to connectivity issues. Solutions, such as creating a backup of the information within the device and improved communication protocols, were integrated into NavegApp’s beta version to prevent future data loss. All the participants could finish at least the first 3 trials for each task, which indicated that the wording of the instructions was clear so the participants with MCI could tackle the cognitive tasks.

### Alpha Testing Stage: Content Validity

The expert panel assessed the relevance and representativeness of gamified cognitive tasks in NavegApp in alignment with the SC model by Jacobs [13]. Table 2 displays Aiken V and modified κ indexes, gauging expert consensus. The results show unanimous agreement, with a 100% consensus per the κ index, supporting task relevance and representativeness. The Aiken V index indicated high agreement (ie, between 96% and 100%) among experts regarding task relevance and representativeness.

**Table 2:** Content validity measures.

Task	Relevance			Representativeness	
	κ	Aiken V (95% CI)	κ		Aiken V (95% CI)
Allocentric spatial navigation (gHGT^a^)	1.00	0.96 (0.80-0.99)	1.00		0.96 (0.80-0.99)
Mental rotation (gMRT^b^)	1.00	0.96 (0.80-0.99)	1.00		0.96 (0.80-0.99)
Short-term visuospatial working memory (gCorsi^c^)	1.00	1.00 (0.86-1.00)	1.00		1.00 (0.86-1.00)

^a^gHGT: gamified version of a hidden goal task.

^b^gMRT: gamified mental rotation task.

^c^gCorsi: gamified Corsi Block-Tapping test.

### Alpha Testing Stage: Digital Ergonomics

Following the recommendations for conducting and analyzing Delphi studies [46], an anonymous report was crafted for each expert, and new data regarding their opinion on the digital ergonomics criteria were collected. No differences were found between the first- and second-round scores on any ergonomic criteria. Scores of digital ergonomics for rounds 1 and 2 are provided in Multimedia Appendix 2. Qualitative recommendations such as changes in the wording of instructions, adequations in the interfaces, gamification elements, and a tutorial for the gHGT were included in the beta version of NavegApp.

NavegApp’s digital ergonomics were measured using a Likert scale of 7 points. Scores for each criterion were determined by averaging the related items. To summarize the perceptions of the expert panel and healthy young adults, median and IQRs were calculated. The median scores for all ergonomic criteria indicated a perception exceeding 75% (ie, >5.25), reflecting a positive evaluation of NavegApp’s digital ergonomics. Table 3 shows minor differences (*r*_rb_ 0.08-0.29) between the expert panel and healthy young adults regarding their perceptions of digital ergonomics. The experts rated NavegApp’s ergonomics slightly higher than healthy young adults, except for the codes significance and orientation criteria. Details of statistical tests are provided in Multimedia Appendix 2.

**Table 3:** Digital ergonomics perception comparison.

Ergonomic criteria	Experts (n=8), median (IQR)	Healthy young adults (n=20), median (IQR)	Rank biserial correlation, *r*_rb_ (95% CI)
Compatibility	6.75 (1.13)	6.00 (2.00)	0.16 (0.01 to 0.48)
Orientation	6.75 (0.13)	7.00 (0.31)	0.25 (0.02 to 0.59)
Load	6.67 (1.84)	6.33 (0.92)	0.08 (0.01 to 0.52)
Adaptability	7.00 (0.00)	7.00 (0.50)	0.19 (0.01 to 0.46)
Consistency	7.00 (0.17)	6.67 (1.08)	0.29 (0.03 to 0.57)
Codes significance	5.50 (0.25)	5.63 (1.75)	0.26 (0.02 to 0.54)
Control	7.00 (0.00)	7.00 (0.25)	0.13 (–0.36 to 0.56)

### Alpha Testing Stage: Difficulty and Agreeableness

**Figure 4:**
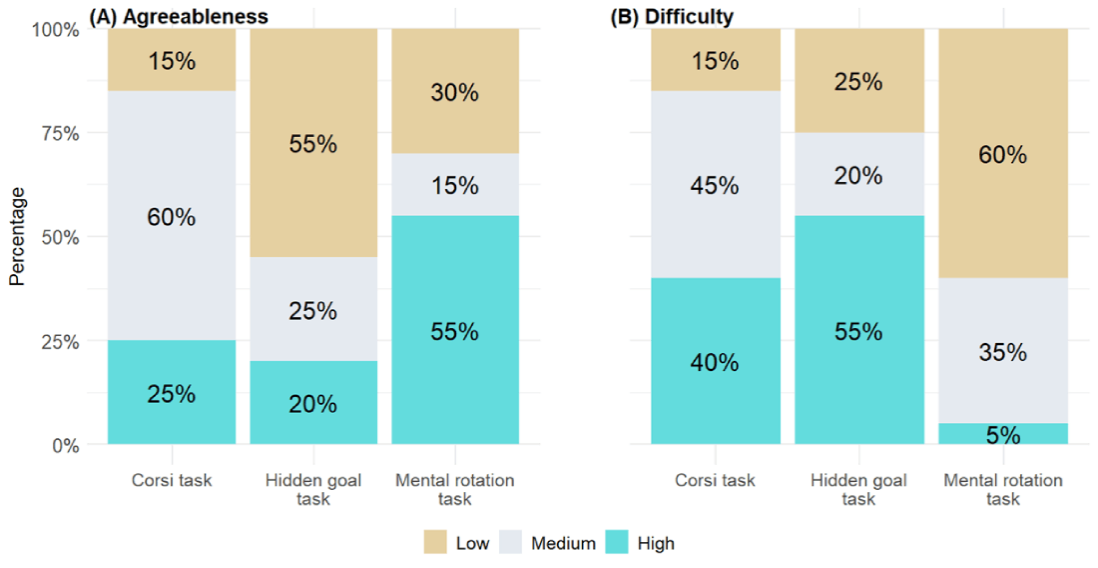
Agreeableness and difficulty perception by task.

Following experts’ suggestions, the healthy young adult sample was asked to order the tasks according to agreeableness and difficulty. Analysis using Cramér V indicated a moderate association between the perception of agreeableness and difficulty for each task (*χ*^2^_4_=16.5; *P*=.002; V=0.37, 95% CI 0.28-0.54). Further examination of the frequency distribution indicated that as tasks were perceived as more difficult, participants found them less agreeable. Figure 4 displays the difficulty (Figure 4A) and agreeableness (Figure 4B) perception by task.

### Beta Testing: Usability Assessment

Table 4 details descriptive statistics and effect size measures used to quantify the differences in participants’ perceptions of usability, as measured by the VG-CSUQ questionnaire components, for the beta version of NavegApp.

**Table 4:** Usability perception.

Usability scores	Caregivers (n=22), median (IQR)	PSEN1-E280A mutation carriers (n=81), median (IQR)	Healthy controls (n=59), median (IQR)	MCI^a^ (n=14), median (IQR)
Usability total score	93.3 (13.6)	94.4 (11.1)	94.4 (10.0)	83.9 (32.2)
System usefulness	91.7 (10.4)	91.7 (16.7)	91.7 (16.7)	70.8 (33.3)
Information quality	96.7 (12.5)	96.7 (10.0)	96.7 (10.0)	88.3 (25.0)
Interface quality	100 (16.7)	100 (0.0)	100 (4.2)	95.8 (25.0)

^a^MCI: mild cognitive impairment.

Rank biserial correlation was used to assess the magnitude of differences in usability perceptions among the groups testing the beta version of NavegApp. The analysis revealed that the sporadic MCI group showed moderate to large differences, with lower scores in overall usability perception (*r*_rb_ 0.26-0.29), system usefulness (*r*_rb_ 0.25-0.37), information quality (*r*_rb_ 0.27-0.34), and interface quality (*r*_rb_ 0.13-0.23) when compared to other groups. In contrast, participants from the caregivers, asymptomatic PSEN1-E280A mutation carriers, and healthy control groups exhibited minor differences (*r*_rb_ 0.00-0.20), indicating a more consistent usability perception. A table detailing the magnitude of these differences for each pair of groups is included in Multimedia Appendix 3.

## Discussion

### Principal Findings

In this study, we detail the development and validation of NavegApp, a serious video game designed for assessing SC components potentially sensitive to early cognitive change in the AD continuum. Our results confirm that NavegApp is a usable and ergonomic SG encompassing cognitive tasks that are representative and relevant for measuring SC. The consensus among experts and positive feedback on digital ergonomics perception and usability from healthy young adults, caregivers, and final users demonstrates that NavegApp offers a user-friendly interface for executing gamified cognitive tasks effectively. Central to the performance of NavegApp is our approach, which integrates recommendations for usability and digital ergonomics assessment, along with validity measurements directly into the SDLC, particularly during the testing phase [33,36,57].

Our results from the alpha testing stage indicated that NavegApp consistently performed well across multiple devices, accurately displaying all stimuli and instructions with minimal data loss. Notably, participants diagnosed with MCI and early-stage dementia could comprehend and complete at least the initial 3 trials for each task, meeting the primary criteria for task comprehension [11]. These findings align with other studies of SGs developed under similar frameworks, which report positive usability metrics from the early stages of software development [35,66,67]. In addition, our results support using the Unity Game Engine as a suitable platform for developing 2D interfaces for cognitive assessment [68]. Although most research using Unity for cognitive assessment has focused on 3D spatial navigation tasks [18,69], its versatility makes it ideal for creating 2D interfaces compatible with various devices, such as tablets and mobile phones [47,68]. This versatility is crucial for deploying digital tasks in underrepresented populations with limited access to advanced technologies or expensive devices.

Designing reliable and valid SGs for cognitive assessment requires a strong foundation in validity and reliability metrics [7]. Analyzing content validity is crucial in this context, as it assesses whether an instrument effectively measures its intended construct [37,61]. For NavegApp, the expert panel demonstrated a high level of consensus, confirming that all tasks included in the SG were relevant and representative of the SC model by Jacobs [13]. Furthermore, qualitative feedback from the panel provided insights into improving the SG’s theoretical foundations and user-interface elements [30,45,46]. This study is the first to include a content validity analysis within the SG development process, setting it apart from other games, primarily focusing on usability [35] and acceptability [67].

Key components of the content validity analysis presented here involved conducting a thorough critical literature review and following the original task protocols to guide the gamification process from the beginning of the SDLC [9,11,46]. Content validity indexes were computed to quantify expert consensus on the relevance and representativeness of various components within the instrument [45,46]. This process aimed to avoid discrepancies in determining the tasks to be included in the SG. In addition, the Delphi method, a structured approach facilitating expert dialogue on complex topics, has proven especially beneficial for gaining insights into content validity and digital ergonomics [46].

Additional considerations, such as potential biases stemming from perceptions of visual stimuli and instructions, were examined using statistical methods during NavegApp’s development alpha testing phase. Our findings revealed an association between agreeableness and difficulty perception, prompting us to randomize level presentations to participants in the SG final version. This ensures unbiased performance and prevents overarching perceptions of the test as difficult or unpleasant [70,71]. According to our findings, design decisions can benefit from a phased evaluation approach, such as the one used in this study, wherein earlier test versions establish a foundational benchmark for assessing subsequent iterations.

While including an expert panel primarily facilitates content validity assessment, their expertise can provide valuable insights into important aspects such as digital ergonomics for building user-friendly interfaces. Integrating digital ergonomics into the SDLC from the alpha stage provides critical information on how design decisions impact the end-user experience [11,44]. Although these principles have been used for developing SG in the context of assessment and training [44,64], there is currently no systematic framework for their inclusion in the SG development process.

To address this gap, our study introduces a self-reported questionnaire that converts digital ergonomics heuristics by Bastien and Scapin [57] into Likert scale items to evaluate 8 fundamental ergonomics criteria recognized as relevant for the design of SG in the context of neurodegenerative diseases [11]. The median scores for these criteria indicated >90% (ie, all scores >6.30) acceptability for NavegApp’s interface, except the codes significance criterion, which scored 79% (ie, 5.50). Code significance refers to the semantic relationship between a term and the actions it represents. Scores obtained from experts and young, healthy adults underscore the need to enhance icons and vocabulary to improve user experience and interactions in the subsequent beta phase testing stage. This multistage approach, incorporating multiple sources of evidence, enriches the current literature on the development of SGs for cognitive assessment in neurodegenerative diseases, which often prioritize aspects such as immersive [38], user-interface interactions [39], and gamification [35].

Moving into the beta testing stage, usability assessment was integrated into the SDLC. Usability questionnaires are particularly valuable during this phase [56], as the qualities of the interface, system, and information provided by the software are crucial for enhancing user interaction efficiency [11,42]. While some SGs for cognitive assessment have demonstrated high acceptance [67,69], gaps remain in examining these aspects comprehensively. To address this, we used the VG-CSUQ, an adaptation of the widely used CSUQ, to gather multifaceted usability data in the SG context. This approach contributed to a nuanced assessment and subsequent adjustments of system features, thereby refining users’ usability perception.

Given the lack of country- or setting-specific normative data, usability percentages offer a proxy for user perceptions [42]. Median scores exceeding 75% across all usability factors indicate that NavegApp is consistently perceived as usable across all tested groups. All groups rated the interface quality and system usefulness highly, with perception rates exceeding 75%, and no differences were found among the groups.

The differences in information quality perception between sporadic MCI and healthy adult groups can be attributed to the higher perceived difficulty of the tasks by those with decreased cognitive functionality. Despite this, the perception remained highly positive (ie, >80%). This finding suggests that digital tools may be more challenging to implement for individuals in the prodromal stage of AD, necessitating additional efforts to enhance their user experience [11]. It underscores the importance of including participants across the A spectrum during development to avoid spectrum bias and ensure the SG is tested beyond a healthy population [7,11].

Our findings have important implications for cognitive assessment theories related to AD, particularly in the context of screening and diagnosis. Cognitive assessment is a cornerstone in the staging of individuals along the AD continuum, as the presence or absence of subjective and objective cognitive decline distinguishes preclinical and prodromal stages of the disease [3]. Traditionally, subjective cognitive concerns and episodic memory are assessed through person-to-person paper-and-pencil neuropsychological tests, which remain the standard for follow-up, screening, and assessment in at-risk populations [3]. However, the emergence of innovative digital tools, such as SGs, presents an opportunity to rethink these methods. SGs can conduct cognitive assessments using engaging and cost-effective methodologies while maintaining psychometric rigor [9]. Although challenges remain in achieving fully remote cognitive screening programs, our results demonstrate the feasibility of developing user-friendly and highly usable interfaces capable of implementing cognitive assessment protocols in a standardized manner. These findings align with and extend existing theories by demonstrating how digital tools can complement traditional approaches, potentially improving accessibility and scalability in cognitive assessments for AD [9,11].

The development of innovative SGs for cognitive assessment, such as NavegApp, offers unique opportunities for early screening and intervention in AD. These tools bring several advantages to clinical practice. First, SGs eliminate the need for specialized, trained personnel during task execution, enabling remote cognitive assessments and expanding access to at-risk populations. Second, by fostering enhanced engagement and motivation, SGs improve data quality in repetitive cognitive tasks compared to traditional paper-and-pencil assessments [35]. Third, SGs provide greater ecological validity by simulating real-world tasks, offering insights into functional cognition in everyday contexts [7]. In addition, these tools reduce costs and mitigate biases arising from individual differences among evaluators, ensuring consistency in data collection [9]. Together, these features position SGs as powerful instruments for large-scale cognitive screening and monitoring, with the potential to support early diagnosis and intervention in clinical settings.

### Limitations

While our results favor NavegApp as a usable tool, it is essential to acknowledge certain limitations. Due to the exploratory nature of the feasibility assessment and the need to collect qualitative data on the performance and perception of potential users, a nonprobabilistic sampling method was preferred for the analyses presented here. However, these sampling methods limit the generalizability of our findings, necessitating further studies to align our results with broader population characteristics [72]. In addition, given the lack of standardized tools for assessing digital ergonomic criteria, we introduced a tailored self-reporting questionnaire grounded in previous recommendations for SG design in neurocognitive disease contexts [11].

However, the psychometric robustness of this tool warrants future evaluation.

Our content validity assessment hinges on a select group of experts meeting specific criteria. Different experts might offer varying degrees of agreement. Finally, while our CSUQ questionnaire adaptation is valuable and represents an effort to develop standardized instruments for assessing the usability of SGs adapted to the Spanish-speaking populations from low- and middle-income countries, it is not exclusive. Alternative instruments, such as the System Usability Scale [44] or Gamefulquest [73], are also suitable options for gauging software and SG usability and user experience.

As group comparisons were a central focus of this study, the imbalance in group sizes, stemming from the nonprobabilistic sampling approach, represents a limitation that warrants consideration. Uneven sample sizes can affect the precision of effect size estimates and the reliability of CIs, potentially introducing bias into group comparisons [72]. Future studies should aim to address this limitation by ensuring balanced sample sizes across groups. Conducting a priori power analyses can help determine the appropriate sample size needed to achieve sufficient statistical power for group comparisons [72]. In addition, using matched sampling methods could further enhance the quality of comparisons by reducing variability and improving the robustness of findings.

Future studies should incorporate additional levels of analysis to refine the development and evaluation of SGs for cognitive assessment. For instance, comparing perceptions and performance across distinct groups using the same software version during the early development phase can help identify potential biases introduced during the gamification process. Conversely, contrasting different software versions at various stages of development can reveal biases stemming from design decisions and modifications to the interface and learning effects associated with repeated use of the SG. To achieve this, longitudinal usability assessments are recommended, enabling the systematic evaluation of changes over time [39].

The preexisting validity evidence of cognitive tasks plays a critical role in shaping the development process, influencing the steps and metrics required for validation and usability. However, a phased approach that collects data on multiple aspects of validity and usability at different stages of development provides richer insights than a singular pilot study. Such an approach facilitates a more comprehensive understanding of task performance and usability dynamics. Finally, future research should investigate how enhanced game design can more effectively target specific cognitive functions. By tailoring tasks to assess nuanced aspects of SC, they could improve their sensitivity for detecting subtle cognitive impairments. This targeted approach may help advance the utility of SGs as scalable tools for early screening and diagnosis of neurodegenerative conditions such as AD.

### Conclusions

As the need for scalable cognitive screening programs and early detection of cognitive decline intensifies, digital neuropsychological alternatives, such as SGs, are emerging as a suitable alternative to traditional methods based on molecular biomarkers. These games offer a viable solution for remote, repeated assessments on a population scale [4,5,33]. Despite advances in the field, there is an urgent need to create digital tools that balance both validity and usability for the cognitive testing of people at risk for developing or living with neurodegenerative diseases, such as AD [4]. In this context, we introduce NavegApp, a valid and user-friendly SG designed for SC assessment, crafted by merging software development principles and measurement properties evaluation [37].

The adoption of SGs in health informatics signifies a strategic shift, addressing the limitations inherent in traditional neuropsychological assessment [5,9,11,33]. This study was conducted in response to the growing call for tools that prioritize user experience and validity and are primed for broad-scale cognitive monitoring. While innovative, our approach emphasizes the crucial role of integrating psychometric validity measures from the outset of SG design. Using a blend of usability evaluations, content validity assessments, and digital ergonomic principles, we aim to set the stage for evolving standardized developmental frameworks. Such frameworks are poised to capitalize on cutting-edge health informatics and psychometrics advancements in digital neuropsychology.

## Appendix

Multimedia Appendix 1 Digital ergonomic questionnaire for serious games design.

Multimedia Appendix 2 Digital ergonomics scores and comparisons from Delphi rounds.

Multimedia Appendix 3 Usability perception comparison among groups.

## Abbreviations

AD: Alzheimer disease
CSUQ: Computer System Usability Questionnaire
gHGT: gamified version of a hidden goal task
MCI: mild cognitive impairment
SC: spatial cognition
SDLC: software development life cycle
SG: serious game
VG-CSUQ: Computer System Usability Questionnaire for videogame
VR: virtual reality

## Data Availability

The data supporting this study’s findings are not publicly available due to privacy and confidentiality concerns. However, the data can be accessed from the corresponding author upon reasonable request.
