# Supplementary material for: Digital Ergonomics of NavegApp, a Novel Serious Game for Spatial Cognition Assessment: Content Validity and Usability Study
Source: JMIR Serious Games. 2025 Apr 2;13:e66167. doi: 10.2196/66167 (PMC12004023; doi:10.2196/66167)
Supplement: Multimedia Appendix 1 [file games_v13i1e66167_app1.docx]

## Multimedia Appendix 1

### Digital Ergonomic Questionnaire for Serious Games Design.

Ergonomic criteria involve design principles aimed at creating effective and user-friendly interfaces. This survey is based on the ergonomic criteria Ben-Sadoun et al. [11] outlined for developing serious games in cognitive assessment for neurodegenerative diseases. Below, you will find a series of statements with corresponding response options. Please indicate your level of agreement with each statement by marking a number from 1 to 7, where one means "Completely Disagree" and seven means "Completely Agree," which best reflects your viewpoint.

| **Ergonomic criteria** | **Criteria Definition** | **Digital Ergonomic Item** |  |
| --- | --- | --- | --- |
|  |  |  |  |
| ***Compatibility.*** | Coincidence between user characteristics and system characteristics. | NavegApp is adapted to the physical characteristics of the target population. |  |
|  |  | NavegApp is adapted to the cognitive characteristics of the target population. |  |
| ***Orientation.*** | Availability to advise, orient, inform and guide users through their interactions with the system so that they always know where they are and what they can do. | NavegApp provides real-time guidance. |  |
|  |  | NavegApp provides feedback on game progression. |  |
|  |  | The information presented in the interface is organized in a way that is easy to understand. |  |
|  |  | The commands in NavegApp are presented simply. |  |
| ***Load.*** | The elements included in the interface should help reduce the cognitive load and increase the efficiency of the dialogue. | The number of commands in NavegApp is sufficient to solve the tasks. |  |
|  |  | The number of motor actions required in NavegApp can be minimized. |  |
|  |  | The information presented in the NavegApp interface is the information needed to solve the tasks. |  |
| ***Adaptability.*** | The ability of the system to behave contextually, according to the needs and preferences of the users. | The actions performed by the player lead him to reach the goal at each level. |  |
|  |  | NavegApp integrates different levels of difficulty in the tasks. |  |
| ***Consistency.*** | How interface design decisions hold in similar contexts and differ in different contexts. | The window titles are located in similar positions. |  |
|  |  | The screen formats in NavegApp are similar. |  |
|  |  | Access to the NavegApp menus is similar. |  |
|  |  | Similar punctuation and wording are used in the different windows. |  |
|  |  | The data entry prompts are displayed in a position similar to other programs. |  |
|  |  | The data entry fields in NavegApp are similar. |  |
| ***Codes significance.*** | The semantic relationship between a term or sign and the actions or objects to which it refers. Codes and names are meaningful when there is a semantic relationship between those codes and the items or actions they refer to. | The NavegApp scenarios are "familiar" to the target population (i.e., the adult population at risk of AD). |  |
|  |  | The images used in NavegApp are easy to understand. |  |
|  |  | The text of the commands in NavegApp is clear and concise. |  |
|  |  | NavegApp uses icons and symbols unknown to the players (i.e., the adult population at risk of AD). |  |
| ***Explicit control.*** | The processing of explicit user actions and the control that users have over the system's processing of their actions. | Tasks in NavegApp start after a specific action by the user (e.g. clicking "Next"). |  |
|  |  | Commands in NavegApp require confirmation for execution (e.g. Confirm progression to the next level). |  |
| ***Error Management.*** | The ability of the system to prevent or reduce errors and recover when they occur. | Fields displaying information are protected. |  |
|  |  | The interface controls accidental user errors. |  |
|  |  | Error messages invite action on the part of the user. |  |
|  |  | The error messages are explicit. |  |
|  |  | Error messages are short but informative. |  |
|  |  | Error messages have neutral phrasing. |  |
|  |  | Users can edit personal information before submitting it. |  |
|  |  | Users can make corrections immediately and directly. |  |
|  |  | After the error, the user only must enter the incorrect information. |  |
