# Supplementary material for: Digital Ergonomics of NavegApp, a Novel Serious Game for Spatial Cognition Assessment: Content Validity and Usability Study
Source: JMIR Serious Games. 2025 Apr 2;13:e66167. doi: 10.2196/66167 (PMC12004023; doi:10.2196/66167)
Supplement: Multimedia Appendix 2 [file games_v13i1e66167_app2.docx]

## Multimedia Appendix 2

### Digital Ergonomics scores and comparisons from Delphi rounds.

The following presents scores from both rounds of content validity. The scores from round one were reflected in round two.

|  | **Round 1** | | **Round 2** | |
| --- | --- | --- | --- | --- |
|  | **Mean (SD)** | **Median [IQR]** | **Mean (SD)** | **Median [IQR]** |
| Compatibility | 12.75 (1.58) | 13.5 [2.3] | 12.75 (1.58) | 13.5 [2.3] |
| Orientation | 26.75 (1.49) | 27 [1.5] | 26.75 (1.49) | 27 [1.5] |
| Load | 18.25 (3.37) | 20 [5.5] | 18.25 (3.37) | 20 [5.5] |
| Adaptability | 13.63 (1.06) | 14 [0.0] | 13.63 (1.06) | 14 [0.0] |
| Consistency | 41.38 (1.06) | 42 [1.0] | 41.38 (1.06) | 42 [1.0] |
| Significance | 20.63 (2.45) | 22 [2.0] | 20.63 (2.45) | 22 [2.0] |
| Load | 12.75 (2.82) | 14 [0.5] | 12.75 (2.82) | 14 [0.5] |
| Rules | 17.25 (2.25) | 17 [4.0] | 17.13 (2.17) | 17 [3.3] |

*Note = SD = Standard Deviation; IQR = Interquartile Range.*

The following table presents the results of statistical comparisons between groups.

| **Ergonomic Criteria** | **Experts (n=8)** | | **Healthy young adults (n=20)** | | **U Mann Whitney Statistical Test** |
| --- | --- | --- | --- | --- | --- |
|  | **Median** | **IQR** | **Median** | **IQR** |  |
| Compatibility | 6.75 | 1.13 | 6.00 | 2.00 | U=96; p= .41; r_rb_=0.16; CI 95%= 0.01,0.46 |
| Orientation | 6.75 | 0.13 | 7.00 | 0.31 | U=57; p= .2; r_rb_=0.25; CI 95%= 0.01,0.62 |
| Load | 6.67 | 1.84 | 6.33 | 0.92 | U=88.5; p= .68; r_rb_=0.08; CI 95%= 0.01,0.48 |
| Adaptability | 7.00 | 0.00 | 7.00 | 0.50 | U=95.5; p= .34; r_rb_=0.19; CI 95%= 0.01,0.47 |
| Consistency | 7.00 | 0.17 | 6.67 | 1.08 | U=108; p= .14; r_rb_=0.29; CI 95%= 0.02,0.55 |
| Significance | 5.50 | 0.25 | 5.63 | 1.75 | U=46; p= .18; r_rb_=0.26; CI 95%= 0.02,0.55 |
| Control | 7.00 | 0.00 | 7.00 | 0.25 | U=79; p= .52; r_rb_=0.1; 3CI 95%= 0.01,0.4 |

*Note. U = U Mann-Whitney statistic value; p = p-value; rrb = Rank biserial Correlation; CI 95% = Confidence Interval at 95%.*
