# Supplementary material for: Digital Ergonomics of NavegApp, a Novel Serious Game for Spatial Cognition Assessment: Content Validity and Usability Study
Source: JMIR Serious Games. 2025 Apr 2;13:e66167. doi: 10.2196/66167 (PMC12004023; doi:10.2196/66167)
Supplement: Multimedia Appendix 3 [file games_v13i1e66167_app3.docx]

## Multimedia Appendix 3

### Usability perception comparison among groups

The following presents the differences among groups in the usability perception metrics.

| **Usability Score** | **Group 1** | **Group 2** | **Rank biserial correlation** | |
| --- | --- | --- | --- | --- |
|  |  |  | **r_rb_** | **95% IC** |
| Usability Total Score | PSEN1-E280A Carrier | Caregivers | 0.13 | [0.01, 0.29] |
|  | PSEN1-E280A Carrier | Healthy Controls | 0.02 | [0.0029, 0.2] |
|  | PSEN1-E280A Carrier | Sporadic MCI | 0.26 | [0.06, 0.43] |
|  | Caregivers | Healthy Controls | 0.13 | [0.0086, 0.33] |
|  | Caregivers | Sporadic MCI | 0.27 | [0.02, 0.57] |
|  | Healthy Controls | Sporadic MCI | 0.29 | [0.06, 0.49] |
| System Usefulness | PSEN1-E280A Carrier | Caregivers | 0.06 | [0.003, 0.23] |
|  | PSEN1-E280A Carrier | Healthy Controls | 0.00 | [0.0024, 0.19] |
|  | PSEN1-E280A Carrier | Sporadic MCI | 0.25 | [0.03, 0.44] |
|  | Caregivers | Healthy Controls | 0.06 | [0.006, 0.26] |
|  | Caregivers | Sporadic MCI | 0.37 | [0.05, 0.66] |
|  | Healthy Controls | Sporadic MCI | 0.28 | [0.04, 0.48] |
| Information Quality | PSEN1-E280A Carrier | Caregivers | 0.01 | [0.004, 0.29] |
|  | PSEN1-E280A Carrier | Healthy Controls | 0.06 | [0.0024, 0.22] |
|  | PSEN1-E280A Carrier | Sporadic MCI | 0.27 | [0.07, 0.47] |
|  | Caregivers | Healthy Controls | 0.18 | [0.0091, 0.4] |
|  | Caregivers | Sporadic MCI | 0.30 | [0.03, 0.6] |
|  | Healthy Controls | Sporadic MCI | 0.34 | [0.11, 0.56] |
| Interface Quality | PSEN1-E280A Carrier | Caregivers | 0.20 | [0.01, 0.4] |
|  | PSEN1-E280A Carrier | Healthy Controls | 0.13 | [0.0064, 0.3] |
|  | PSEN1-E280A Carrier | Sporadic MCI | 0.23 | [0.02, 0.45] |
|  | Caregivers | Healthy Controls | 0.12 | [0.0064, 0.35] |
|  | Caregivers | Sporadic MCI | 0.13 | [0.0087, 0.46] |
|  | Healthy Controls | Sporadic MCI | 0.18 | [0.009, 0.43] |

*Note = MCI = Mild Cognitive Impairment; r_rb_ = Rank biserial correlation*
